# Differential Effects of Migration and Deportation on HIV Infection among Male and Female Injection Drug Users in Tijuana, Mexico

**DOI:** 10.1371/journal.pone.0002690

**Published:** 2008-07-30

**Authors:** Steffanie A. Strathdee, Remedios Lozada, Victoria D. Ojeda, Robin A. Pollini, Kimberly C. Brouwer, Alicia Vera, Wayne Cornelius, Lucie Nguyen, Carlos Magis-Rodriguez, Thomas L. Patterson, for Proyecto El Cuete

**Affiliations:** 1 School of Medicine, University of California San Diego, La Jolla, California, United States of America; 2 Patronado Pro-COMUSIDA A.C., Tijuana, Mexico; 3 Department of Political Science, University of California San Diego, La Jolla, California, United States of America; 4 Centro Nacional para la Prevención y el Control del VIH/SIDA, Mexico City, Mexico; 5 Department of Veterans Affairs Medical Center, San Diego, La Jolla, California, United States of America; Instituto de Pesquisa Clinica Evandro Chagas, FIOCRUZ, Brazil

## Abstract

HIV prevalence is rising, especially among high risk females in Tijuana, Baja California, a Mexico-US border city situated on major migration and drug trafficking routes. We compared factors associated with HIV infection among male and female injection drug users (IDUs) in Tijuana in an effort to inform HIV prevention and treatment programs. IDUs aged ≥18 years were recruited using respondent-driven sampling and underwent testing for HIV, syphilis and structured interviews. Logistic regression identified correlates of HIV infection, stratified by gender. Among 1056 IDUs, most were Mexican-born but 67% were born outside Tijuana. Reasons for moving to Tijuana included deportation from the US (56% for males, 29% for females), and looking for work/better life (34% for females, 15% for males). HIV prevalence was higher in females versus males (10.2% vs. 3.5%, p = 0.001). Among females (N = 158), factors independently associated with higher HIV prevalence included younger age, lifetime syphilis infection and living in Tijuana for longer durations. Among males (N = 898), factors independently associated with higher HIV prevalence were syphilis titers consistent with active infection, being arrested for having ‘track-marks’, having larger numbers of recent injection partners and living in Tijuana for shorter durations. An interaction between gender and number of years lived in Tijuana regressed on HIV infection was significant (p = 0.03). Upon further analysis, deportation from the U.S. explained the association between shorter duration lived in Tijuana and HIV infection among males; odds of HIV infection were four-fold higher among male injectors deported from the US, compared to other males, adjusting for all other significant correlates (p = 0.002). Geographic mobility has a profound influence on Tijuana's evolving HIV epidemic, and its impact is significantly modified by gender. Future studies are needed to elucidate the context of mobility and HIV acquisition in this region, and whether US immigration policies adversely affect HIV risk.

## Introduction

Tijuana is the largest city on the Mexican-U.S. border in the state of Baja California, Mexico, with an estimated population of 1.4 million persons [1]. Internal migration from Southern Mexico and Central America has led Tijuana to experience one of the highest population growth rates in Latin America. In addition to being a major transportation route for migrants headed for the United States (U.S.), Tijuana is situated on a major drug trafficking route. Approximately 95% of heroin entering the U.S. west of the Mississippi River and 95% of all methamphetamine entering the U.S. is of Mexican origin, and 70% of South American cocaine is transported through Mexico [2], [3]. Tijuana has the largest number of drug users per capita and an estimated 10,000 injecting drug users (IDUs) [4]. Tijuana also has a thriving *zona roja* [red light zone], where the city's estimated 9,000 female sex workers (FSWs) can legally work [5].

Baja California continues to have the highest cumulative AIDS incidence among Mexico's 32 states, second only to the federal district of Mexico City [6]. In recent years, HIV prevalence and incidence has risen markedly among some high risk populations in Tijuana [7]. where approximately half of the state's inhabitants reside. In 2006, HIV prevalence among FSWs rose to 6% in Tijuana [5], an increase from ∼1% prior to 2002 [8]. HIV prevalence was 12% among FSWs who inject drugs in Tijuana; nearly half of whom had at least one of four active sexually transmitted infections (STI), including HIV [9]. In a large study of pregnant women in Tijuana, HIV prevalence was 1% and 6% among pregnant women who reported using illicit drugs [10]. Based on these and other available data from 2005, it was estimated that as many as one in 125 persons aged 15–49 in Tijuana was HIV-infected [11], suggesting that the city's HIV epidemic had progressed from low-level to concentrated. The estimated male:female ratio of HIV cases approached 3∶1, compared to 4∶1 in Mexico overall [6].

We recently reported that HIV prevalence among IDUs in Tijuana has remained low at 3% between 2005 and 2006–2007 [12], [13], but was significantly higher among female versus male IDUs [9], [12]. Since data from other countries suggests that female IDUs have greater overlap in their sexual and drug using networks relative to male IDUs [14], [15], [16], [17] we hypothesized that sexual and social relationships would factor prominently as HIV-related risk factors among females. We compared correlates of HIV infection among male and female IDUs in Tijuana, and assessed a range of potential risk factors at the individual, network and environmental levels that could lead to new avenues for intervention.

## Methods

### Study Population

During 2006 and 2007, IDUs were recruited in Tijuana into a prospective study of behavioral and contextual factors associated with HIV, syphilis and TB infections [12]. Eligibility criteria included: being age ≥18 years; having injected illicit drugs within the past month; ability to speak Spanish or English; being able to provide written informed consent; and having no plans to permanently move out of the city in the following 18 months. The protocol was approved by the Institutional Review Boards of the University of California, San Diego and Tijuana General Hospital.

Participants were recruited by indigenous outreach workers using respondent-driven sampling (RDS), a sampling technique that enables the calculation of less biased estimates of HIV prevalence and risk behaviors [18]. A diverse group of IDU “seeds” (heterogeneous by age, gender and neighborhood) were selected and given uniquely coded coupons to refer their peers.

### Study Instrument

Participants completed an interviewer-administered survey eliciting information on sociodemographic, behavioral and contextual characteristics, which was developed based on responses from an earlier study of IDUs in Tijuana [13], [19]. Sociodemographic questions included income, living arrangements and migration history (place of birth, number of years lived in Tijuana). Among persons who were not born in Tijuana, we asked reasons for moving to the city which included seeking employment, a ‘better life’, drugs, family reasons, deportation or other reasons (which participants were asked to specify). Reasons reported under ‘other’ were examined to enable potential re-coding where appropriate (e.g., planned versus unplanned moves). Deportation history was based on self-report since we could not verify the occurrence of legal removal proceedings in the U.S.

Participants were asked about their lifetime and current (past 6 months) sexual behaviors and drug use. Network-related questions included having an IDU sex partner, number of injection partners, number of IDUs in their social network and whether their sex partners injected drugs. Sexual behaviors of interest included unprotected sex with regular, casual and client partners of the same or opposite sex. Participants were also asked whether they had ever been arrested and if so, whether they had ever been arrested for possessing used or unused/sterile syringes, or for having track marks (i.e., injection stigmata), since these reasons were commonly reported as reasons for arrest in a prior qualitative study [4].

### Laboratory Testing

The “Determine”® rapid HIV antibody test was used to detect the presence of HIV antibodies (Abbott Pharmaceuticals, Boston, MA). Reactive samples were confirmed using an HIV-1 enzyme immunoassay and immunofluorescence assay. Syphilis serology was conducting using the rapid plasma reagin (RPR) test (Macro-Vue, Becton Dickenson, Cockeysville, MD, USA); RPR-positive samples were confirmed using the *Treponema pallidum* particle agglutination assay (TPPA) (Fujirebio, Wilmington, DE, USA). Syphilis titers ≥1∶8 were considered to be consistent with active infection, whereas the remainder of positive specimens was considered to reflect lifetime, rather than current, infection. Specimen testing was conducted at the San Diego County Health Department. Participants testing positive for HIV or other sexually transmitted infections (STIs) were referred to the Tijuana municipal health clinic for free care.

### Statistical Analysis

We first compared male and female IDUs in terms of sociodemographic variables, social influence, risk behaviors/exposures and environmental/structural exposures, followed by a comparison of HIV-positive and HIV-negative IDUs, stratified by gender. Continuous outcomes were examined using t-tests and Wilcoxon Rank Sum tests for differences in group distributions for normally and non-normally distributed variables, respectively. Binary outcomes were examined using the Pearson Chi-square or Fisher's exact test.

Univariate and multivariate logistic regressions were performed to identify factors associated with HIV-positive serostatus, stratified by gender. In multivariate regressions, a manual procedure was used whereby all the variables that had attained a significance level <10% in univariate models were considered for inclusion. We also considered variables that had been previously independently associated with high risk behaviors or HIV infection [12], [20]. Planned moves were considered as having moved to Tijuana for employment, a better life or for family reasons, whereas unplanned moves were related to deportation. The likelihood ratio test was used to compare nested models, using a significance level of 5%. All two-way interactions were explored. For ease of interpretation, we present age and number of years lived in Tijuana in terms of ten year increases, and number of persons with whom they injected and number of arrests for track marks for every five persons or arrests.

As described previously [12], we explored potential effects of RDS on our estimates using the RDS Analysis Tool (version 5.6.0, October 2006, Cornell University) and WinBUGS (version 1.4.1, Imperial College and Medical Research Council, UK, 2004). Odds ratios and 95% confidence intervals produced by the RDS analyses were compared to our multivariate logistic regression models. Since no significant differences between the RDS-adjusted and unadjusted model were identified, unadjusted models are presented.

## Results

### Gender Differences in HIV and associated Risks

Of 1,056 IDUs, 896 (86%) were male and 157 (14%) were female. Compared to males (Table 1), females were younger (median 34 versus 37 years, p<.001) and a higher proportion were married or in common-law relationships (50% versus 28%, p<0.001) or had an IDU sex partner (11% versus 2%, p<0.001). In terms of drug use history, females tended to have injected drugs for shorter durations than males (median: 12 versus 15 years, p<0.001), and a lower proportion reported ever engaging in receptive needle sharing (45% versus 61%, p<0.001).

**Table 1:** Characteristics of Male and Female IDUs with and without HIV infection in Tijuana, Mexico; 2006–2007.

Baseline Characteristics	Males				Females			
	HIV+	HIV−	Total	P-value	HIV+	HIV−	Total	P-value
	N = 31	N = 865	N = 896		N = 16	N = 141	N = 157	
***Sociodemographics***								
Median age (IQR)	37 (31–44)	37 (32–43)	37 (32–43)	0.86	26 (24–32)	35 (29–41)	34 (28–41)	0.001
Median (IQR) # years of education completed	7 (6–9)	7 (6–9)	7 (6–9)	0.72	6 (6–8)	8 (6–10)	8 (6–10)	0.11
Speaks some English	20 (65%)	420 (49%)	440 (49%)	0.10	3 (19%)	66 (47%)	69 (44%)	0.04
Average monthly income≥3000 pesos	23 (79%)	591 (70%)	614 (70%)	0.31	11 (73%)	85 (61%)	96 (62%)	0.41
Married/common-law	11 (35%)	240 (28%)	251 (28%)	0.41	7 (44%)	72 (51%)	79 (50%)	0.61
***Social Influence***								
Sex partner is an IDU*	0 (0%)	12 (2%)	12 (2%)	1.00	2 (14%)	11 (11%)	13 (11%)	0.65
Median (IQR) number of IDUs in social network	70 (40–200)	70 (40–140)	70 (40–140)	0.34	75 (56–100)	60 (40–120)	60 (40–120)	0.48
Median (IQR) # hours spent daily on the street*	12 (12–18)	10 (7–12)	11 (8–13)	0.001	12 (9–12)	8 (4–12)	9 (5–12)	0.02
Median (IQR) # people usually injected with*	3 (2–5)	2 (1–3)	2 (1–3)	0.002	2 (1–4)	2 (1–2)	2 (1–2)	0.61
Ever forced to have sex	1 (3%)	8 (1%)	9 (1%)	0.27	4 (27%)	39 (28%)	43 (28%)	1.00
***Individual Behaviors/Risks***								
Median (IQR) duration (years) of injection	13.9 (5.8–21.4)	15.0 (9.7–22.0)	15.0 (9.5–22.0)	0.38	5.0 (2.6–10.5)	12.9 (6.8–19.8)	12.1 (6.1–19.0)	0.01
Any receptive needle sharing*	17 (55%)	531 (61%)	548 (61%)	0.46	8 (50%)	63 (45%)	71 (45%)	0.79
Shared injection paraphernalia≥half the time*	1 (3%)	82 (9%)	83 (9%)	0.35	0 (0%)	7 (5%)	7 (4%)	1.00
Used new/sterile needle≥half the time*	16 (52%)	370 (43%)	386 (43%)	0.36	6 (38%)	77 (55%)	83 (53%)	0.29
Obtained syringes from needle exchange program*	5 (83%)	141 (71%)	146 (72%)	0.68	5 (56%)	28 (70%)	33 (67%)	0.45
Ever had unprotected sex with HIV+ person	4 (13%)	16 (2%)	20 (2%)	0.004	2 (13%)	1 (1%)	3 (2%)	0.03
Syphilis titer≥1∶8	7 (23%)	47 (5%)	54 (6%)	0.001	5 (31%)	20 (15%)	25 (16%)	0.14
Positive for syphilis antibodies	9 (29%)	97 (11%)	106 (12%)	0.007	11 (69%)	46 (33%)	57 (36%)	0.01
Ever traded sex in exchange for money, drugs, goods or shelter	2 (6%)	154 (18%)	156 (17%)	0.14	9 (64%)	75 (54%)	84 (55%)	0.58
Ever had sex with someone of the same gender	6 (19%)	230 (27%)	236 (26%)	0.42	5 (33%)	42 (30%)	47 (30%)	0.77
Ever had HIV test	6 (19%)	319 (37%)	325 (36%)	0.06	7 (44%)	97 (69%)	104 (66%)	0.05
High perceived risk of HIV infection	21 (70%)	383 (45%)	404 (46%)	0.01	10 (63%)	47 (33%)	57 (36%)	0.03
***Structural/Environmental Factors***								
Born in Mexico	30 (96.8%)	861 (99.5%)	891 (99.4%)	0.16	14 (87.5%)	123 (87.2%)	137 (87.3%)	1.00
Born outside of Baja California	27 (87%)	577 (67%)	604 (67%)	0.02	11 (69%)	91 (65%)	102 (65%)	1.00
Ever traveled to the U.S.	28 (90%)	689 (80%)	717 (80%)	0.17	4 (25%)	99 (70%)	103 (66%)	<.001
Deported from the U.S.	22 (71%)	355 (41%)	377 (42%)	0.001	2 (13%)	34 (24%)	36 (23%)	0.37
Moved to Tijuana was planned	6 (21%)	287 (44%)	293 (43%)	0.02	8 (80%)	77 (67%)	85 (68%)	0.50
Median # years lived in Tijuana (IQR) per 10 years	0.8 (0.4–1.5)	1.5 (0.5–3.0)	1.5 (0.5–3.0)	0.03	1.6 (1.0–2.6)	1.2 (0.4–2.5)	1.3 (0.5–2.5)	0.19
Homeless*	8 (26%)	126 (15%)	134 (15%)	0.12	0 (0%)	8 (6%)	8 (5%)	1.00
Normally injected drugs outside*	11 (35%)	229 (26%)	240 (27%)	0.30	0 (0%)	9 (6%)	9 (6%)	0.60
Normally injected drugs at shooting gallery*	13 (42%)	369 (43%)	382 (43%)	1.00	1 (6%)	19 (13%)	20 (13%)	0.70
Ever been arrested	28 (90%)	762 (88%)	790 (88%)	1.00	12 (75%)	112 (80%)	124 (80%)	0.74
Ever arrested for carrying used needle/syringe¥	14 (52%)	352 (46%)	366 (46%)	0.70	3 (25%)	32 (29%)	35 (28%)	1.00
Median (IQR) number of times arrested for carrying used needle/syringe¥	2 (0–10)	0 (0–5)	0 (0–5)	0.41	0 (0–2)	0 (0–1)	0 (0–1)	0.80
Ever arrested for having track marks¥	21 (78%)	498 (65%)	519 (66%)	0.22	7 (58%)	63 (56%)	70 (56%)	1.00
Median (IQR) number of times arrested for having track marks¥	3 (1–15)	3 (0–10)	3 (0–10)	0.45	2 (0–9)	1 (0–6)	2 (0–7)	0.75
Median (IQR) number of times in jail/prison	2 (1–3)	2 (1–4)	2 (1–4)	0.93	1 (0–1)	0 (0–2)	0 (0–2)	0.85
Ever injected in jail**	15 (58%)	401 (61%)	416 (61%)	0.84	5 (56%)	33 (52%)	38 (53%)	1.00

* Last 6 months.

** Among those ever incarcerated (N = 682 for males and N = 72 for females).

¥ Among those ever arrested (N = 790 for males and N = 124 for females).

Significantly higher proportions of females had a syphilis infection consistent with active (16% versus 6%, p<0.001) or lifetime infection (36% versus 12%, p<0.001) compared to males. Higher proportions of females had ever been forced to have sex (28% versus 1%, p<0.001) or had traded sex (55% versus 17%, p<0.001). Although a higher proportion of females had previously had an HIV test relative to males (66% versus 36%, p = <0.001), a lower proportion of females considered themselves at high HIV risk (36% versus 46%, p = 0.02).

The proportion of female and male IDUs who were born outside of Baja California was similar (approximately two thirds); males and females had also lived in Tijuana for similar durations. However, a higher percentage of females indicated that their move to Tijuana was planned compared to males (68% vs. 43%, p<0.0001). Females were more likely to report having moved to Tijuana for a job or better life (34%) or family reasons (16%), compared to males (15% and 10% respectively). Compared to females, males were more likely to have ever traveled to the U.S. (80% vs. 66%, p<0.001) and to report having arrived in Tijuana as a result of deportation from the U.S. (42% versus 23%, p<0.001); in fact, deportation was the most commonly cited reason for moving to Tijuana among males.

Compared to females, higher proportions of males reported being homeless (15% versus 5%, p<0.001), injecting mostly outside (27% versus 6%, p<0.001) or in a shooting gallery (i.e., a location where people inject in groups and rent/buy used syringes; 43% versus 13%, p<0.001). Compared to females, males were more likely to have ever been arrested (88% vs. 80%, p = 0.01), were more frequently arrested for carrying used syringes (46% vs. 28%, p<0.001), and were somewhat more likely to report being arrested for having injection stigmata (i.e., ‘track marks’; 66% vs 56%, p = 0.06).

Overall, the unweighted HIV prevalence (i.e. unadjusted for RDS) for the sample was 4.5%, but was higher in females than males (10.2% vs. 3.5%, p = 0.001; data not shown). The RDS-adjusted HIV prevalence was lower at 3.0%, but remained higher in females relative to males (5.4% vs. 2.4%, p<0.001).

### Correlates of HIV Infection among Females

In univariate analyses, HIV-positive females were younger and less likely to speak English compared to HIV-negative females (Tables 1 and 2), but factors relating to social influence were not associated with significantly higher HIV infection. In terms of individual-level exposures, HIV-positive females had been injecting for shorter durations, and were more likely to report ever having sex with an HIV-positive person compared to HIV-negative females, although numbers were small. A higher proportion of HIV-positive females had evidence of lifetime (but not active) syphilis infection and a higher proportion perceived themselves to be at high HIV risk, compared to HIV-negative females. HIV-positive females were marginally less likely to have ever had an HIV test compared to HIV-negative females (44% vs. 69%, p = 0.05), suggesting that the majority of HIV-infected females were not previously aware of their HIV-serostatus.

**Table 2:** Factors associated with HIV infection among Male and Female IDUs in Tijuana.

*Baseline Characteristics*	Males		Females	
	Odds Ratio	95% Confidence Interval	Odds Ratio	95% Confidence Interval
***Sociodemographics***				
Age (per 10 years)γ	0.93	0.60–1.45	0.28	0.12–0.64
Number of years of education completed γ	0.97	0.87–1.08	0.89	0.77–1.02
Speaks English¥ γ	1.93	0.91–4.07	0.26	0.07–0.96
Average monthly income≥3000 pesos	1.65	0.67–4.11	1.75	0.53–5.77
Married/common-law	1.43	0.68–3.03	0.75	0.26–2.11
***Social Influence***				
Sex partner is an IDU	0.97	0.06–16.8	1.41	0.28–7.14
Number of IDUs in social network (per 5 people)	1.00	1.00–1.00	1.00	0.98–1.02
Number of hours spent daily on the street* ¥	1.10	1.03–1.16	1.07	0.98–1.17
Number of people usually injected with (per 5 people)* ¥	1.21	1.03–1.42	1.53	0.41–5.70
Ever forced to have sex	3.57	0.43–29.5	0.94	0.28–3.13
***Individual Behaviors***				
Duration of injection (years) γ	0.99	0.95–1.03	0.90	0.83–0.98
Any receptive needle sharing*	0.76	0.37–1.57	1.24	0.44–3.48
Shared injection paraphernalia≥half the time*	0.32	0.04–2.36	0.54	0.03–9.96
Used new/sterile syringes≥half the time*	1.43	0.70–2.92	0.50	0.17–1.45
Obtained syringes from needle exchange program*	1.08	0.14–8.19	0.54	0.12–2.35
Ever had unprotected sex with HIV+ person¥ γ	7.85	2.46–25.1	21.2	1.80–250
Syphilis titer≥1∶8¥ γ	5.30	2.16–13.0	2.66	0.83–8.47
Positive syphilis antibodies ¥ γ (i.e., lifetime exposure)	3.24	1.45–7.24	4.54	1.49–13.8
Ever traded sex for money, drugs, goods or shelter	0.32	0.08–1.35	1.54	0.49–4.82
Ever had sex with someone of the same sex	0.66	0.27–1.63	1.17	0.38–3.62
Ever had an HIV test¥ γ	0.41	0.17–1.01	0.35	0.12–1.01
High perceived risk of HIV infection¥ γ	2.84	1.29–6.27	3.33	1.14–9.73
***Structural/Environmental Factors***				
Born in Mexico¥	0.14	0.02–1.28	1.02	0.21–4.88
Born outside of Baja California¥	3.36	1.16–9.69	1.21	0.40–3.68
Ever traveled to the U.S. γ	2.38	0.72–7.93	0.14	0.04–0.46
Deported from the U.S. ¥	3.51	1.60–7.72	0.45	0.10–2.08
Moved to Tijuana was planned ¥	0.34	0.14–0.86	1.97	0.40–9.75
Number of years lived in Tijuana (per 10 years) ¥	0.68	0.50–0.93	1.15	0.81–1.64
Homeless* ¥	2.04	0.89–4.66	0.48	0.03–8.63
Normally injected drugs outside*	1.53	0.72–3.24	0.42	0.02–7.60
Normally injected drugs at shooting gallery*	0.97	0.47–2.01	0.43	0.05–3.43
Ever arrested	1.26	0.38–4.22	0.75	0.22–2.50
# arrests for having track marks (per 5 arrests) ¥	1.10	1.00–1.22	1.10	0.86–1.42
# arrests for carrying used syringes (per 5 arrests)	1.13	0.97–1.32	0.79	0.36–1.76
# times in jail/prison (per 5 times)	0.76	0.41–1.39	0.51	0.12–2.17
Ever injected in jail	0.87	0.39–1.92	1.14	0.28–4.63

* refers to last six months.

¥ p-value≤0.10 for males.

γ p-value≤0.10 for females.

HIV-positive females were less likely to have traveled to the U.S., but were no more likely to have been born outside of Baja California, to have moved to Tijuana due to deportation, or to have planned to move to Tijuana compared to HIV-negative females. Although not statistically significant, having lived in Tijuana for a longer period of time was associated with an elevated odds of HIV infection (OR: 1.15 per 10 years; 95% CI: 0.81–1.64), an association that became significant after controlling for age (Adjusted OR [AdjOR]: 1.89 per 10 years; 95% CI: 1.10–3.24; data not shown).

In a multivariate model (Table 3), factors independently associated with HIV infection among females included older age (AdjOR: 0.18 per 10 year increase; 95% CI: 0.08–0.42), lifetime syphilis infection (AdjOR: 4.5; 95% CI: 1.40–14.51) and living in Tijuana for longer durations. Specifically, for every ten years that female IDUs lived in Tijuana, their odds of testing HIV-positive increased 81% (AdjOR: 1.81 per 10 year increase; 95% CI: 1.12–2.94). No significant interactions were observed in this model.

**Table 3:** Factors independently associated with HIV infection among Male and Female IDUs in Tijuana, Mexico.

Variable	Adjusted Odds Ratio for Males (95% CI)	Adjusted Odds Ratio for Males (95% CI)	Adjusted Odds Ratio for Females (95% CI)
	(Model 1)	(Model 2)	
Age (per 10 years)	–		0.18 (0.08–0.42)
Active syphilis Titer≥1∶8	6.24 (2.43–16.05)	5.61 (2.16–14.55)	–
Syphilis positive TPPA	–		4.50 (1.40–14.51)
Number of different people usually injects with* (per 5 people)	1.25 (1.07–1.46)	1.28 (1.10–1.48)	–
Number of arrests for track marks (per 5 arrests)	1.12 (1.00–1.25)	1.10 (0.97–1.24)	–
Number of years lived in Tijuana (per 10 year increase)	0.65 (0.46–0.93)	–	1.81 (1.12–2.94)
Deported from the U.S	–	4.00 (1.67–9.44)	–

* last 6 months.

### Correlates of HIV Infection among Males

**Figure 1:**
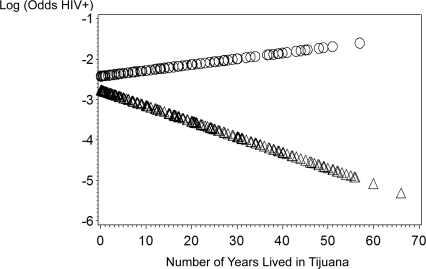
Gender: ΔΔΔMale ΟΟΟ Female.

Among males, no sociodemographic variables were associated with HIV infection (Tables 1 and 2). In terms of social influence, spending more time on the street and injecting with a larger group were both associated with HIV infection in univariate models. Ever having sex with an HIV-positive person, having evidence of a current or lifetime syphilis infection, and having a high perceived risk of acquiring HIV were associated with higher odds of HIV infection. In terms of environmental/structural influences, having been arrested for having track marks, being born outside of Baja California, having been deported from the U.S., not having planned to move to Tijuana and living in Tijuana for shorter durations were associated with higher odds of HIV infection. In particular, the association between duration of time lived in Tijuana and the odds of HIV infection was opposite for males versus females (Figure 1). A formal test of the interaction between gender and number of years lived in Tijuana regressed on HIV infection was statistically significant (p = 0.03).

In a multivariate model (see Model 1, Table 3), HIV-positive males were significantly more likely to have syphilis antibody titers consistent with active infection (AdjOR: 6.24; 95% CI: 2.43–16.05). At the network level, odds of HIV-positivity increased 25% for every five additional injection partners in the prior six months. At the environmental/structural level, the odds of testing HIV-positive increased by 12% for every five arrests for having track marks, although this association was of borderline significance. Males who had lived in Tijuana for shorter durations were also more likely to test HIV-positive; for every 10 years lived in Tijuana, odds of HIV-positivity decreased by 35% (AdjOR: 0.65; 95% CI: (0.46–0.93).

We further explored the association between duration of time lived in Tijuana and HIV infection, since this relationship was unanticipated and opposite of that observed among females. When deportation from the U.S. was offered into Model 1, the variable reflecting duration of time lived in Tijuana was no longer significant (AdjOR: 0.83; 95% CI: 0.55–1.24), whereas deportation was significantly associated with HIV infection (AdjOR: 2.95; 95% CI: 1.03–8.46). After removing duration of time lived in Tijuana from the model, deportation was associated with a four fold increased odds of HIV infection among males (AdjOR: 4.0; 95% CI: 1.67–9.44; see Model 2, Table 3), suggesting that deportation explained the association of higher odds of HIV infection among male newcomers to Tijuana. In comparing the Akaike Information Criterion statistic (AIC) between Model 1 and Model 2, as recommended by the literature [21], the latter was smaller, indicating that Model 2 was a better fit.

## Discussion

Our study of HIV risk factors among male and female drug injectors in a Mexico-U.S. border city yielded two significant findings. First, prevalence of HIV infection and potential risk factors differed markedly by gender. Second, geographic mobility had a differential effect on the risk of HIV infection among male and female IDUs. These findings have important implications for HIV prevention, diagnosis and treatment on both sides of the Mexico-U.S. border.

We observed nearly a three-fold higher HIV prevalence among female IDUs compared to male IDUs in Tijuana, as reported previously [12]. HIV prevalence among female IDUs was similar to that reported recently among FSWs who inject drugs in Tijuana [9]. However, only half of the female IDUs in the present study reported trading sex for money, drugs or other commodities, and trading sex was not associated with HIV infection. On the other hand, the strong association we observed between history of syphilis and HIV infection among female IDUs was similarly observed among FSWs in Tijuana [5]. Baja California has the highest cumulative incidence of syphilis among all Mexican states, at 12 per 100,000 [6]. Since syphilis is a known cofactor of HIV transmission [22] and was the only risk factor in common among female and male IDUs, syphilis diagnosis and eradication should be an integral component of HIV prevention in this region.

Contrary to our hypothesis, we observed no association between HIV prevalence and social influence among female drug injectors, such as having an IDU sex partner, injecting in groups, or having more IDU peers. While our findings support other studies that demonstrate significant gender differences in HIV risk factors and the importance of sexual behaviors as drivers of HIV infection [23], [24], [25], our study differs from U.S. studies which suggest that sexual and injecting networks have a significant bearing on HIV acquisition among female IDUs [15], [16], [17]. Since the number of female IDUs in this study was relatively small, these relationships warrant further study.

Although two thirds of our sample was born outside of Baja California, a greater percentage of female IDUs indicated that their migration to Tijuana was planned relative to males. This may help explain the protective association we observed with female newcomer status and lower odds of HIV-positivity, since planned moves may occur in the context of greater stability. Females who moved to Tijuana in search of employment or due to family reasons may have had more supportive social ties that buffered against HIV risk behaviors such as needle sharing and unprotected sex with multiple partners. Foreign birthplace has been associated with lower rates of substance use among persons of Mexican heritage living in the U.S.[27], [28], [29], which has been referred to as the ‘healthy migrant’ effect [30].

It is also possible that female newcomers initially had less exposure to HIV because of traditional sex roles that expect Mexican women to care for their family and home, and limit their sexual experiences to a marital relationship [31], [32]. In a prior qualitative study among IDUs in Tijuana and Ciudad Juarez (another Mexico-US border city, adjacent to El Paso TX), females tended to buy and inject drugs in their homes in the company of a small, trusted circle of peers, whereas males were more likely to buy and inject drugs in public spaces with large numbers of people, including strangers [33]. In an earlier cross-sectional study of IDUs in Tijuana and Ciudad Juarez, females had smaller injection networks than male IDUs [13],which may have limited their exposure to HIV, albeit for a short time. Our data suggest that as the duration spent in Tijuana increases, protective effects associated with being a female newcomer are outweighed by other, unexplained factors that warrant closer examination.

An unexpected finding was that among males, newcomers were more likely to be HIV-infected. Living in Tijuana for *longer* periods was associated with lower HIV prevalence, a relationship that was starkly opposite that of females. Over half of male newcomers had been deported from the U.S., a figure that was more than three-fold higher than an earlier study of male IDUs in Tijuana who reported having been deported from the U.S. [19]. In the present study, deportation was independently associated with four-fold increased odds of HIV-positivity among males, whereas there was no association among females. In a previous study of primarily male drug injectors recruited from shooting galleries in Tijuana, newcomer status was associated with receptive needle sharing [20], but this association may have been confounded since the role of deportation was not explored.

The causal implications of our findings are unclear, but we propose two plausible explanations. The first possibility is that deportation is a marker for a high risk subgroup of Mexican male migrants, which would suggest that mobility–rather than deportation—creates social and structural conditions that predispose to HIV acquisition. In 2005, one fifth of IDUs in Tijuana had traveled to the U.S. in the previous year [19]. Tijuana's pool of migrants in transit to the U.S. or recently expelled from the U.S. continues to grow robustly, despite recent border enforcement enhancements. With the median income gap between Mexico and the U.S. being the largest between any two contiguous developed and developing countries, strong economic incentives continue to draw hundreds of thousands of Mexicans to the U.S. each year. Moreover, the vast majority of people in Mexican high-emigration towns have one or more relatives living in the United States, which promotes migration for family reunification [32]. Tijuana and San Diego share the busiest land border crossing in the world, with 45.9 million north-bound legal border crossings in 2006 alone [35]. In 2006, the U.S. Border Patrol made more than 80,000 apprehensions in the Tijuana sector [36]. A recent study of Oaxacan undocumented migrants to the U.S. found that, on their most recent trip to the border, 72% had entered in the San Diego/Tijuana area.

Trans-border mobility has been identified as an important risk factor in the acquisition of several communicable diseases, including HIV [37], [38], [39], [40], ostensibly due to high risk behaviors following disintegration of family support networks, sudden changes in the cultural environment, homelessness, and poverty. Aral suggests that migration can change the “sexual structure” at the place of destination, for example, by introducing young people without sex partners into new social networks where HIV prevalence may be higher than the sending community [41]. Some migrants may be risk-takers among whom there may be a greater sense of anonymity [42], [43], [44], [45], [46], promoting riskier behaviors in the absence of ‘structural and normative environmental checks and balances on sexual behaviour’ [41].

Among drug users, newcomers are more likely to engage in needle sharing or attend shooting galleries [45], [46], [48], perhaps out of the need to obtain drugs, needles or social acceptance. Studies of Mexican migrants suggest that stressors such as continued unemployment, homelessness and family separation can also promote high risk behaviors including unprotected sex with sex workers and other men, as well as sharing injection equipment when injecting illicit drugs, vitamins or antibiotics [42], [43], [47], [49]. These social and structural processes may predispose migrants to greater vulnerability of acquiring HIV in the U.S through unprotected sex or drug use; evidence supporting both pathways was observed in our study.

An alternate explanation for our finding is that deportation from the U.S. is the precipitating factor leading to social upheaval, loss of social ties and income, homelessness, and possibly other destabilizing conditions which lead to engagement in high risk behaviors and HIV acquisition after expulsion. In our earlier, smaller study comparing IDUs who involuntarily moved to Tijuana due to deportation versus those who moved to Tijuana for other reasons, the former were less knowledgeable about HIV and HCV risks, and were less likely to have had an HIV test or drug treatment [19]. In that study, deportees also had different patterns of drug use, suggesting that newcomers may change the ‘structure of drug using networks’ at the place of destination, in a similar manner described by Aral and colleagues [41].

It is common for undocumented migrants who are apprehended, either in the immediate border area or in the U.S. interior, to be transported to the U.S.-Mexico border where they are repatriated with few, if any, belongings or sources of assistance. For many, Tijuana becomes their new temporary or long-term ‘home.’ Further studies are needed to determine whether circumstances surrounding deportation heighten vulnerability to HIV infection, and if so, whether there are specific interventions of U.S. border enforcement and Mexican repatriation policies/procedures that could interrupt the HIV transmission chain. For example, a promising new Mexican government program to provide support (e.g., temporary shelter, food, clothing, transportation, medical attention) for Mexican nationals recently deported from the U.S. is being implemented in Tijuana, Nogales and possibly other Mexican border cities.

This study confirmed our earlier finding that frequent arrest for track marks was independently associated with HIV infection [12], but in the present study this associated persisted only among males. Although in the same direction for females, this association may not have attained significance due to low statistical power. However, it is also possible that this is a true gender difference. We and others have speculated that these and other policing practices, such as arresting IDUs for carrying syringes, may pressure drug injectors to borrow used syringes from their peers or to rent them from shooting galleries, which can heighten their HIV risks [50], [51], [52], [53], [54]. On the other hand, these men might be subject to greater social marginalization due to homelessness, disheveled appearance, scarring from track marks, tattoos, or newcomer status, making them more vulnerable to arrest[12].

Since this study is cross-sectional, we cannot elucidate a causal role of mobility on the risk of HIV infection. Similarly, we cannot determine whether HIV was more likely to be acquired in the U.S. or Mexico. We may have underestimated the number of drug injectors who were deported, since most persons apprehended by the U.S. Department of Homeland Security (∼90%) are “voluntarily” (vs. forcibly) returned to their country of origin[55]. However, misclassification would tend to have attenuated the associations we observed between deportation and HIV infection towards the null. While we cannot confirm that participants began injecting drugs before being deported, male deportees had been living in Tijuana an average of 8 years, but had been injecting drugs an average of 16 years.

Further study is needed to prospectively examine the role of migration and displacement as risk factors versus protective factors for HIV infection in various social, geographic and cultural contexts, taking into account potentially modifying influences such as gender. These studies should examine the role of other factors that may have a direct or indirect impact on the risk of HIV infection in this region, such as involvement in gangs and drug trafficking. Future investigations should incorporate both qualitative and quantitative methodologies to illuminate potential causal mechanisms and avenues for intervention.

Even in the absence of additional data, our findings and the accumulated evidence point to the need for supportive programs targeted at migrants, deportees and other displaced persons on both sides of the U.S.-Mexico border. Prevention programs should include culturally and linguistically appropriate educational materials on HIV and STIs, availability of free condoms and sterile syringes, access to free HIV/STI testing with pre- and post-test counseling and drug abuse treatment. Treatment programs should integrate HIV/STI treatment and ensure that once initiated, treatment can continue as medically indicated regardless of location, mobility, or immigration status. Political will and sustained binational cooperation are needed to ensure that policies dealing with immigration control and health services are working in tandem, rather than at odds.
